# Robotic Manipulation under Harsh Conditions Using Self‐Healing Silk‐Based Iontronics

**DOI:** 10.1002/advs.202102596

**Published:** 2021-11-05

**Authors:** Mengwei Liu, Yujia Zhang, Yanghong Zhang, Zhitao Zhou, Nan Qin, Tiger H. Tao

**Affiliations:** ^1^ State Key Laboratory of Transducer Technology Shanghai Institute of Microsystem and Information Technology Chinese Academy of Sciences Shanghai 200050 China; ^2^ School of Graduate Study University of Chinese Academy of Sciences Beijing 100049 China; ^3^ Center of Materials Science and Optoelectronics Engineering University of Chinese Academy of Sciences Beijing 100049 China; ^4^ 2020 X‐Lab Shanghai Institute of Microsystem and Information Technology Chinese Academy of Sciences Shanghai 200050 China; ^5^ School of Physical Science and Technology ShanghaiTech University Shanghai 200031 China; ^6^ Institute of Brain‐Intelligence Technology Zhangjiang Laboratory Shanghai 200031 China; ^7^ Shanghai Research Center for Brain Science and Brain‐Inspired Intelligence Shanghai 200031 China; ^8^ Center for Excellence in Brain Science and Intelligence Technology Chinese Academy of Sciences Shanghai 200031 China

**Keywords:** gesture/object recognition, human–machine interface, silk‐based iontronics, skin electronics/iontronics

## Abstract

Progress toward intelligent human–robotic interactions requires monitoring sensors that are mechanically flexible, facile to implement, and able to harness recognition capability under harsh environments. Conventional sensing methods have been divided for human‐side collection or robot‐side feedback and are not designed with these criteria in mind. However, the iontronic polymer is an example of a general method that operates properly on both human skin (commonly known as skin electronics or iontronics) and the machine/robotic surface. Here, a unique iontronic composite (silk protein/glycerol/Ca(II) ion) and supportive molecular mechanism are developed to simultaneously achieve high conductivity (around 6 kΩ at 50 kHz), self‐healing (within minutes), strong stretchability (around 1000%), high strain sensitivity and transparency, and universal adhesiveness across a broad working temperature range (−40–120 °C). Those merits facilitate the development of iontronic sensing and the implementation of damage‐resilient robotic manipulation. Combined with a machine learning algorithm and specified data collection methods, the system is able to classify 1024 types of human and robot hand gestures under challenging scenarios and to offer excellent object recognition with an accuracy of 99.7%.

## Graphical abstract

This work presents a silk‐based iontronic film which is designed to be simultaneously conductive, self‐healable, antifreezing, and antiheating, based on the developed unique material composition (silk protein/glycerol/Ca(II) ion) and dynamic molecular mechanism. Furthermore, when coupled with a specified machine learning algorithm, the approach permits accurate human/robotic gesture identification across over 1024 classes with high accuracy in harsh environment.

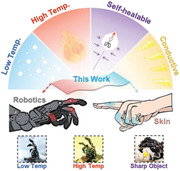

## 1 Introduction

Intelligent human–machine interfaces (HMI), in the simplest definition, include any device or software that allows users to actuate a machine and obtain reliable feedback interactively and intelligently.^[^ ^1^, ^2^, ^3^, ^4^, ^5^, ^6^ ^]^ One of many approaches, the use of hand/body gestures as human‐side input is intuitive and ubiquitous for real‐time high‐fidelity robotic actuation, including but not limited to remotely controlling a robotic hand to grab objects, conducting clinical operations, and performing emergency rescues.^[^ ^7^, ^8^, ^9^, ^10^, ^11^, ^12^, ^13^ ^]^

Current methods for collecting human‐side input and monitoring robot‐side actuation are entirely disparate and not compatible with one another; human‐side input often uses flexible strain sensors to detect irregular and dynamic body movements, while robot‐side feedback directly depends on motor positions to differentiate steps of a continuous action without alternative access.^[^ ^5^, ^14^, ^15^ ^]^ However, the efficacy of this solely motor‐based feedback approach is severely reduced when a mechanical malfunction occurs in sightless or challenging conditions, such as a fire scenario, snow burying, and sharp obstacle cutting. Adopting human‐side strain sensors to monitor robot‐side actuation has been employed as a general approach to overcome this issue. Nevertheless, this type of unified method requires a sensor with capabilities that are suitable for both human and robotic facets, including high‐fidelity sensing, human‐friendly, and flexible composition, ease of mounting on robotics, robustness in harsh environments, while being environmentally friendly and cost‐competitive for broad fabrication.

**Figure 1:**
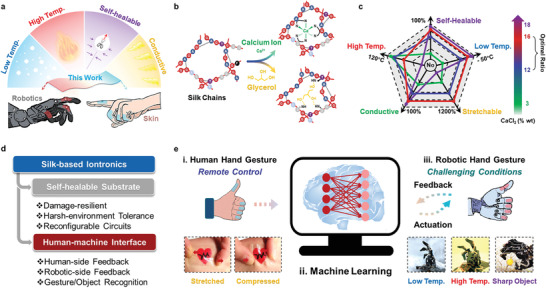
Schematic of multifunctional silk‐based iontronics for HMI and robotic manipulation. a) Schematic illustration showing the major capabilities of silk‐based iontronics. b) Schematic diagram demonstrating the dynamic interactions of glycerol‐induced plasticization and calcium‐induced metal–ligand bonding within the silk chain network. c) Adjusting calcium ion concentrations to tune self‐healing, conductivity, mechanical strength, and temperature tolerance of silk‐based iontronics. d) Proposed framework addressing the main purposes and major features of silk‐based iontronics. e) Schematic illustration showing the intelligent human–machine interaction system, which acquires human hand gestures for the remote control of a robotic hand i), information processing by a machine learning ANN ii), and gesture/object recognition at the robotic side under challenging conditions iii). Inset: Photographs of iontronic substrate mounted on joints of human and robotic hands in various harsh conditions, including harsh temperatures and cutting by sharp obstacles.

In this article, we report a silk‐based iontronic system that can be used on both human skin and robots as a general approach for reliable robotic manipulation under harsh conditions. The silk‐based iontronic film is designed to be simultaneously conductive, self‐healable, antifreezing, and antiheating, based on the developed unique material composition (silk protein/glycerol/Ca(II) ion) and dynamic molecular mechanism (**Figure** **1**a). Compared to the silk‐based substrates in other researches, our work has achieved a simultaneous combination of multiple crucial characterizations without compromising the eco‐friendliness of silk materials (**Table** **1**). Importantly, when coupled with a specified machine learning algorithm, our approach permits accurate human/robotic gesture identification across over 1024 classes, recoverable robotic interaction under challenging temperatures and irrespective of external damage, and grabbed‐object recognition with an accuracy of 99.7%.

**Table 1:** Comparison of different silk‐based iontronics and polymers. In this work, the concentration range of Ca(II) ion is 12–16 wt%, with a fixed glycerol concentration of 5 wt%. RH stands for relative humidity. RT stands for room temperature

Composition	Stretchability	Temperature [°C]	Self‐healing	Conductivity	Application	Reference
Silk/Ca(II)	>200%	RT	NA	Yes (by Au nanotroughs)	Transfer of stretchable electronics	^[^ ^20^ ^]^
Silk/Glycerol	≈200%	RT	NA	NA	Biomedical applications	^[^ ^21^ ^]^
Silk/Graphene/Ca(II)	70–90%	0–50	Yes	Yes (by Graphene)	Epidermal electronics	^[^ ^23^ ^]^
Silk/Ca(II)	>600%	−30–80	Yes	Yes (0.02–6.66 mS cm^−1^)	Temperature sensing	^[^ ^24^ ^]^
Silk/Ca(II)	600% (RH 50%)	NA	Yes	Yes (0.97–1.96 mS cm^–1^)	Flame‐retardant	^[^ ^25^ ^]^
Silk/Ca(II)	400%	RT	NA	Yes (by Au coating)	Stretchable Electrodes	^[^ ^38^ ^]^
Silk/Glycerol/Ca(II)	1000% (RH 50%)	−40–120	Yes	Yes (0.01–11.1 mS cm^−1^)	Gesture/objects recognition under harsh conditions	This work

## 2 Results

The iontronic composite is a ternary system composed of silk fibroin as the framework, glycerol as the plasticizer, and Ca(II) ion as the modifier (Figure 1b). Silk is one of the strongest natural polymers, and it has demonstrated promising applications in skin electronics and wearable sensors.^[^ ^16^, ^17^, ^18^, ^19^, ^20^ ^]^ The facile modifications of silk allow robust strong adhesion to various materials and uneven surfaces, enabling usage on human skin and machine surfaces in this work.^[^ ^19^ ^]^ Notably, Ca(II) ions and glycerol have been used separately with silk in previous works, but the performance in both cases was limited (Table 1). For example, sole glycerol modification could increase mechanical and antiheating capabilities, yet was unable to achieve conductivity or antifreezing.^[^ ^19^, ^21^, ^22^ ^]^ On the other hand, despite endowing self‐healing and remediating other aforementioned deficiencies, Ca(II) ions also impose instability under high temperatures (> 100 °C) or in humid environments due to its strong coordination with labile water molecules. ^[^ ^17^, ^23^, ^24^, ^25^ ^]^ Therefore, our work combines the advantages of glycerol and Ca(II) ions, realizing the synergistic effects of these two additives for the first time.

Specifically, when the final iontronic film is formed (see the Experimental Section), glycerol molecules act as plasticizers in the protein hydration to replace incorporated water, which is needed by Ca(II) ions;^[^ ^19^ ^]^ thus, Ca(II) ions form metal–ligand bonds (chelation) with the silk chains, competing with glycerol in a dynamic balance.^[^ ^26^, ^27^ ^]^ The resultant iontronic film maintains excellent ionic conductivity (around 6 kΩ at 50 kHz), self‐healing properties (within minutes), strong stretchability (around 1000%), high strain sensitivity and transparency, and universal adhesiveness across a broad working temperature range (−40–120 °C, Figure 1c; and Figures S1 and S2, Supporting Information). In addition, adjusting the ratio of Ca(II) ions to glycerol allows modulation of the properties of silk‐based iontronics, as shown in Figure 1c, obtaining performance to be optimized for different working scenarios. A defined concentration range of Ca(II) ions (12–16 wt%) and a fixed glycerol concentration (5 wt%) were selected as the optimal proportions for all‐around performance in subsequent analysis. In order to determine the protein conformation with different compositions, we make Fourier‐transformed infrared spectroscopy characterizations of the silk‐based iontronic film (Figure S3 and Note S3, Supporting Information).

Due to these merits, silk‐based iontronic film can be used in various applications, including as a self‐healable substrate, in reconfigurable circuit components, and for building an HMI system (Figure 1d). These robust capabilities play dominant roles in differentiating subtle changes in human and robotic gestures, as well as in the restoration of substrate/circuit/system damage caused by sharp obstacles and/or harsh temperatures in the environment (Figure 1e). Thanks to these advances and the assistance of a machine learning artificial neural network (ANN), silk‐based iontronics can satisfy the needs of both human and robotic sides for intelligent HMI, even under challenging conditions.

**Figure 2:**
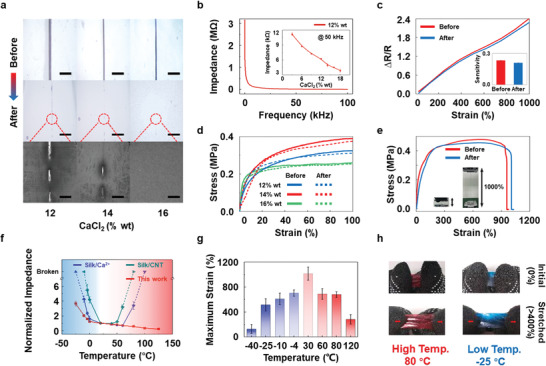
Characterization of electrical and mechanical properties of silk‐based iontronic film. a) Photographs and SEM images of the self‐healing process of silk‐based iontronics with different Ca(II) ion concentrations (12, 14, 16 wt%). The red dashed line represents the magnified region. Scar bar, 1 mm, 500 µm. b) The impedance response curve of silk‐based iontronics with 12 wt% Ca(II) ion concentration. Inset: The impedance at 50 kHz as a function of l Ca(II) ion concentration. c) Strain sensing of silk‐based iontronics before and after self‐healing. Inset: Sensing sensitivity barely changes before and after self‐healing. d) Tensile stress–strain curves of silk‐based iontronics with different Ca(II) ion concentrations (12, 14, 16 wt%) before and after self‐healing. e) The stretchable performance of silk‐based iontronics (14 wt% of Ca(II) ion) before and after self‐healing. Inset: Photographs of a sample with initial length of 1 cm (left) and after being stretched 10 times longer (right). f) Normalized impedance‐to‐temperature responsive curves of different silk‐based films, including that described in this work, silk/Ca(II) and silk(hydrogel)/CNT. g) The maximum strain of silk‐based iontronics in different temperatures. h) Silk‐based iontronic film maintains robust stretchability at high (80 °C) and low (−25 °C) temperatures.

In the ternary material system, Ca(II) ions work mainly as ionic conductors and bonding agents, contributing to self‐healing and conductivity in the iontronic film. As shown in optical and scanning electron microscope (SEM) photographs in **Figure** **2**a, iontronic films with a higher concentration of Ca(II) ions heal faster and more flawlessly at cutting sites (see the Experimental Section). Meanwhile, conductivity of the iontronic film also increases due to the increase in contained ions (Figure 2b). Notably, our iontronic film demonstrated stable mechanical and electrical performance before and after self‐healing (Figure 2c–e; and Figure S4 and Note S4, Supporting Information), guaranteeing the steadiness of strain sensors when contacting with sharp obstacles. As shown in Figure 2c, after the film is scratched and self‐healed, the film could maintain similar stretchable performance and has the same maximum strain compared to which before self‐healing. Thus, the result proves a great self‐healing ability of our silk‐based iontronics. Based on the above mentioned properties, the silk‐based iontronic film could be used as strain sensors in harsh environment. As a strain sensor, in a bounded rectangle iontronic film with area *A* and length *l* carrying a uniform current, the ionic resistance *R* can be simplified as

(1) $$R=R_{0}\frac{l}{l_{0}}\frac{A_{0}}{A}$$

where *R*_0_, *A*_0_, *l*_0_ are the initial resistance, area and length of the iontronic film, respectively. When the iontronic film is stretched, the length *l* is elongated while the average cross‐section area *A* decreases simultaneously, leading to an increased resistance.

Furthermore, previous works have confirmed that environmental factors, such as relative humidity, affect the elasticity modulus of films: the stretchability rises in proportion to the relative humidity (RH);^[^ ^25^ ^]^ therefore, by controlling the environment variables (50% relative humidity), large stretchability (1000%) can be achieved with a Ca(II) ion concentration of 14 wt%. Moreover, the mechanical properties of this iontronic film are tunable by adjusting material proportions, ensuring strong compatibility with large deformations (Figure 2d). For the iontronic film used on the robotic hand, we chose to use silk/glycerol/Ca(II) composite with 14 wt% Ca(II) ions concentration in order to achieve the best stretchability.

In addition to expanding the applicable low‐temperature range with Ca(II) ion content, silk‐based iontronics also demonstrate enhanced antiheating capability as a result of the presence of glycerol molecules, resulting in a broader temperature‐tolerance range. As shown in Figure 2f, the silk/Ca(II) composite could not maintain conductivity in high temperature (higher than 50 °C) and the silk/CNT hydrogel composite could only be conductive in a relative narrow temperature range (20–60 °C). ^[^ ^23^, ^24^ ^]^ Compared with other composites, such as silk/Ca(II) and silk(hydrogel)/CNT, our iontronic film retains relatively constant impedance under harsh temperatures. Furthermore, our iontronic film also exhibits robust stretchability across a temperature range of −25–80 °C (Figure 2g,h), with an operating range of −40 to 120 °C. The excellent mechanical properties and the broad temperature tolerance range of the film largely depend on the silk/glycerol/Ca(II) composite.

In the silk/glycerol/Ca(II) composite, glycerol molecules act as the plasticizer to replace the incorporated water in protein hydration and to form intensified hydrogen bonds with the peptide matrix, resulting in the initial stabilization of *α*‐helical structures in the films, as opposed to random coil or *β*‐sheet structures (Figure S3, Supporting Information). The formation of these strong hydrogen bonding interactions can enhance energy dissipation during the stretching process and thus increase the flexibility of iontronics film. Ca(II) ions can significantly lower the freezing point of water and thereby can significantly enlarge the using temperature range. Besides, with plentiful hydrogen bonds between glycerol and water molecules in our silk/glycerol/Ca(II) composite, the water evaporation rate could be diminished and result in a maintain of stretchability in high temperature (around 700% at 80 °C).^[^ ^19^, ^24^ ^]^

The compelling adjustable properties of our iontronic film enable applications to strain sensors, electronic/iontronic circuits, and system‐level HMI in various challenging situations. For instance, based on specific characterizations, self‐healable silk‐based iontronic films can serve as tunable components in different reconfigurable devices and systems, which can then be triggered by external stimuli and change physical or output electrical signals to meet desired requirements (Figures S5–S7 and Notes S1 and S2, Supporting Information).^[^ ^28^, ^29^, ^30^, ^31^ ^]^

**Figure 3:**
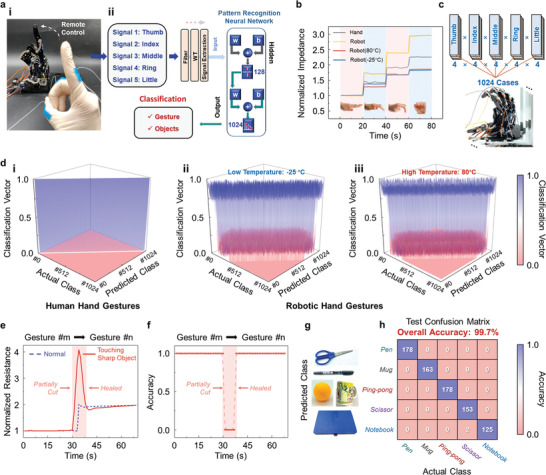
Machine‐learning assisted reliable human–robot interaction under harsh conditions. a) i) Photograph showing remote control of the robotic hand by a human hand. ii) Schematic illustration of data processing scheme and machine learning ANN for gesture/object recognition. b) Sensing of different gestures by human and robotic hands at both high (80 °C) and low (−25 °C) temperatures. c) Data collection of human and robotic hands for each of 1024 gesture types. d) Recognition accuracy of 1024 gesture types for human hand i) and robotic hand in low ii) and high iii) temperatures. e) Normalized impedance variation during gesture change (m to n) with partial cut amid. f) Corresponding recognition accuracy in the process of e). g) Photographs of five different objects. h) Confusion matrix of object recognition.

The previously described silk‐based iontronic devices, including self‐healing strain sensors and reconfigurable circuit components, provide a foundation for an intelligent HMI system used for remote control of a robotic hand and for gesture/object recognition in various challenging conditions. As shown in the process diagram of **Figure** **3**a, after collecting data from strain sensors mounted on both human and robotic hand joints (Figures S8 and S9, Supporting Information), a specified pattern recognition ANN was used for information processing, enabling accurate data classification.^[^ ^19^, ^32^, ^33^ ^]^

The stable electrical properties and high sensitivity of silk‐based iontronics used in strain sensors ensure accurate sensing of various degrees of joint bending and are suitable for use in temperatures as harsh as −25 and 80 °C (Figure 3b). Importantly, operation in these temperatures is likely to cause the robot to malfunction without reliable feedback; our solution provides a general robust monitoring method to solve this issue. By distinguishing the four bending states of each finger, the gesture datasets for human and robotic hands can be established (Figure 3c; and Figure S11, Supporting Information). Due to the robust electrical performance and broad‐range temperature tolerance of the system, gesture classifications for a human hand at room temperature and a robotic hand at both low (−25 °C) and high temperatures (80 °C) can be achieved with high accuracy, realizing a dependable gesture‐based information transmission system appropriate for challenging environments (Figure 3d).

Scratches or cuts caused by surrounding sharp obstacles are often incurred by the robotic hand during operation. Therefore, the self‐healing capability of our strain sensor is vital for continuous and stable detection in harsh environments. Figure 3e indicates that when the mounted iontronic strain sensor is partially cut by an object during gesture changing in an operation task, output signal is fully recovered within 15 s. Consequently, any disturbance in recognition accuracy vanishes swiftly, allowing damage‐resilient gesture feedback (Figure 3f). Furthermore, the ability to accurately measure the curvature degree of the robotic hand fingers also enables precise recognition (99.7%) of grabbed objects of various shapes and sizes (Figure 3g,h). During the experiment, various objects were placed on the table while robotic hand was allowed to grasp the objects at a fixed movement trajectory. This shape‐dependent object recognition is straightforward and can be easily exploited for use with various daily objects, including pens, mugs, ping‐pong balls, scissors, and notebooks.

## 3 Conclusion

By using innovative silk based iontronics, we have reported a general method for the robust recognition of human/robotic hand gestures in robotic actuation and intelligent HMI that is capable of withstanding challenging conditions (e.g., harsh temperatures and cutting by sharp obstacles). Due to intrinsic molecular balancing between glycerol and Ca(II) ions, this green material system attains the versatile capabilities of robust stretchability, self‐healing, broad‐range temperature tolerance, and stable conductivity. These merits facilitate the development of iontronic sensing components and the implementation of damage‐resilient robotic manipulation. Combined with a machine learning algorithm and specified data collection methods, our system is capable of classifying 1024 type of hand gestures made by both humans and robots under challenging scenarios and of recognizing objects with an accuracy of 99.7%.

## 4 Experimental Section

### Silk Solution Preparation

Silkworm silk fibroin proteins were prepared using established purification protocols.^[^ ^34^, ^35^ ^]^ Bombyx mori cocoons were boiled for 30 min in aqueous 0.02 m Na_2_CO_3_ (Sigma‐Aldrich, USA), then rinsed for 3 × 30 min in distilled water to remove Na_2_CO_3_ and sericin. Degummed cocoons were allowed to dry for more than 12 h and subsequently dissolved in 9.3 m LiBr (Sigma‐Aldrich, USA) solution at 60 °C for 4 h. The solution was dialyzed for 2 d in distilled water using Slide‐A‐Lyzer dialysis cassettes (Molecular weight cut‐off, MWCO 3500, Pierce, USA). The solution was centrifuged for 2 × 20 min at 18 000 rpm. The concentration was determined by measuring the volume of solution and the final dried weight.

### Materials and Fabrication of Silk‐Based Iontronic Film

All chemicals were used directly without further purification. The following substances were purchased from Aladdin: calcium chloride, anhydrous (CaCI_2_, C110769), and glycerol (C_3_H_8_O_3_, G116213). During the process of fabricating the silk/Ca(II) ion/glycerol iontronic substrate, purified silk fibroin solution was first mixed with glycerol at a weight ratio of 5%, followed by gentle mixing by a commercial shaker. Next, anhydrous calcium chloride was added at weight ratios of 3–18% (Figure 1c). Then, the mixture was treated by ultrasound at room temperature for 10 min to obtain a well‐dispersed solution. Finally, the blended solution was stored at 4 °C. The blended silk/Ca(II) ion/glycerol solution was sequentially applied to a clean PDMS substrate using a pipette (500 µL solution, substrate size of 3 × 4 cm^2^). Samples were cured for 12 h in an environmental chamber (LichenTech, China) to control relative humidity and temperature, and the silk‐based iontronic substrate was formed with a thickness of around 30 µm. Finally, fabrication methods, such as laser cutting, inkjet printing and mask patterning, were employed for subsequent treatments and modification of the iontronic films (Figure S10, Supporting Information).

### Electrical and Mechanical Characterization

Stress–strain curves were obtained using a CMT4204 mechanical tester (SUST, China). Tensile tests were conducted for more than three samples for each condition at a speed of 1 mm min^−1^. For mechanical measurements at low/high temperatures, samples were placed in a testing chamber and maintained at a certain temperature while tensile tests were conducted (Figure S10, Supporting Information). Electrical properties of the iontronic films were measured using a semiconductor parameter analyzer (Keithley 4200‐SCS, Tektronix). For electrical testing at harsh temperatures, iontronic films were attached to a glass sheet and connected to the analyzer with a silver/silver chloride/copper wire. Fixed films were placed in the oven or refrigerator for real‐time measurement.

### Self‐Healing Experiments

The iontronic film was cut in half with a knife, and the two parts were placed in an environmental chamber as close as possible to one another along the shear line. The temperature was set to 25 °C, and the humidity was set to 50% for 3 min without any external intervention. Of note, the two portions completely merged into a single entity with no obvious scratches remaining. The healed iontronic films can sustain repeated bending and stretching without cracking (Figure S4, Supporting Information). In addition, optical microscopy (EX20, Sunny, China) and SEM (Hitachi S4800) were used to monitor the self‐healing process of the iontronic films (Figure 2a).

### Reconfigurable Circuit Design

To demonstrate the circuit tunability allowed by silk‐based iontronic components, the ability of the iontronics was verified to hold and regulate outputs dynamically. An amplifying circuit was composed of a UA741 precision operational amplifier, two commercially purchased fixed resistors and one self‐healing iontronic resistor. Additionally, a frequency modulation circuit was composed of three commercially purchased fixed resistors, two commercially purchased capacitors and two self‐healing iontronic resistors. During the experiment, electrical signal was supplied by an SA‐SG030 signal generator, and the measurement of resistors was performed by a digit multimeter (Agilent34410A).^[^ ^36^ ^]^ Output voltage and current were measured using a digital oscilloscope (HMO3002 Series). Finally, one piezoelectric circuit was built, which contained one commercially purchased fixed resistor, one self‐healing iontronic resistor and one poly(1,1‐difluoroethylene)‐based piezoelectric nanogenerator, for testing the stable conductivity of iontronic films as wires.^[^ ^37^ ^]^

The fabricated silk‐based iontronic films can be integrated in circuits and act as various components. As shown in Figure 3, different circuits were designed to measure the electrical performance of iontronics as self‐healing wires and circuit components. During the experiment, iontronic films were cut to different extents, including partially and completely. Then, the damaged iontronic film samples were allowed to self‐heal, and the output signal of these circuits was recorded.

### Dataset Collection

After mounting the iontronic strain sensors on the joints of both human and robotic hands, the impedance of each strain sensor was measured at various finger curvature degrees (0°, 30°, 90°, 120°). In this way, datasets of 1024 gesture types for both humans and robots were built. Subsequently, the robotic hand would be exposed to different challenging environments, including low temperatures (−25 °C), high temperatures (80 °C) and damage by surrounding sharp obstacles. Under these conditions, the output signal values of the mounted strain sensors were recorded during the process of remote control by a human hand. Next, the information obtained by the strain sensors was processed via neural network to achieve gesture/object recognition. A custom strain dataset containing 4000 samples distributed into 1024 types of hand gestures was built. Each sample consists of five groups of output resistance data captured from five strain sensors on the joints of the robotic hand.

### ANN Architecture and Design

The core algorithm was written in Python (3.6.4) invoking functions from MATLAB (R2019a). In this article, scaled conjugate gradient backpropagation was chosen as the training function based on tradeoffs between speed and accuracy. In total, five features (one for each finger) were extracted and set as one input vector for classification by the supervised ANN. The ANN with 128 hidden layers and 1024 output classifications, each representing one specific gesture was programed. The input training database was randomly divided into 3:1:1 for training, validation, and testing.

In detail, 3 features were extracted to represent each finger, including real‐time strain‐related normalized impedance, average impedance over 1 s, and corresponding standard deviation; hence, totally 15 features covering 5 fingers were extracted and set as one input vector for the supervised ANN to classify. Then, the scaled conjugate gradient backpropagation as the training function considering the tradeoff between speed and accuracy was chosen. The number of hidden neurons is set as 128 to efficiently complete the input vector (15 features). The ANN with 1024 output classifications, which were labeled as different hand gestures covering 4 states for each finger, was programed. The classification accuracy improves with the number of input data and the ANN with 4000 vectors of data obtained from both human and robotic hands under various environment conditions was trained. The input training database is randomly divided into 3:1:1 for training, validation and testing. Such data amount provides a comprehensive training database of the gesture states for ANN (Figure S12, Supporting Information).

### Statistical Analysis

All experiments were conducted with a minimum of *N* = 5 for each data point; and the data obtained were expressed as the mean standard deviation. Statistical analysis of the data was performed the using SPSS 18.0. The error bars in all figures indicate standard errors.

## Conflict of Interest

The authors declare no conflict of interest.

## Supporting information

Supporting Information

## Data Availability

Research data are not shared.
