# Supplementary material for: Robotic Manipulation under Harsh Conditions Using Self‐Healing Silk‐Based Iontronics
Source: Adv Sci (Weinh). 2021 Nov 5;9(2):2102596. doi: 10.1002/advs.202102596 (PMC8805592; doi:10.1002/advs.202102596)
Supplement: Supplementary file 1 — Supporting Information [file ADVS-9-2102596-s001.pdf]

SUPPLEMENTARY INFORMATION

Supplementary Notes:

1. Self-healable conductive wires

Design For establishing a system-level HMI used for gesture/object recognition in harsh circumstance, self-healing components and recoverable circuits are crucial for maintaining stable physical properties of the system and gaining reliable testing results. Due to the steady electrical and mechanical performance before and after the self-healing process, silk-based iontronics can be used as conductive wires in the circuits, resulting in a superior damage resilient ability of sensing system while being scratched by the surrounding sharp obstacles. As a proof-of-concept, the piezoelectric circuit in Figure S5 can maintain the same output after the iontronic wire being cut and then healing together, showing a potential of the circuit to resist external damages.

2. Components for reconfigurable circuits

On top of damage resistance, self-healable silk-based iontronic film can also serve as tunable components in different reconfigurable circuits, leading to adjustable circuit properties (Figure S6 and S7). As shown in the inset photographs and results, amplitude and frequency of the output signals can be modulated through the cutting and healing process of the iontronic components, suggesting strong potential for a wide gamut of applications (e.g., modular electronics, self-assembling robots, and customizable consumer electronics).

3. Composition of the materials

We make supplementary FTIR characterizations of the silk-based iontronic film in order to determine the protein conformation with different compositions. In principle, glycerol molecules act as the plasticizer to replace the incorporated water in protein hydration and to form intensified hydrogen bonds with the peptide matrix, resulting in the initial stabilization of α-helical structures

in the films, as opposed to random coil or β-sheet structures (Figure S3 (a)). Meanwhile, Ca(II) ions form metal-ligand bonds (chelation) with the silk chains, competing with glycerol in a dynamic balance, introducing extensible structures in silk to improve the stretchability (Figure S3 (a)). Furthermore, as shown in Figure S3 (b), when the concentration of Ca(II) ions increases, the stiff β-sheets decreases and the number of extensible secondary structures increases, such as random coils, resulting in an improved self-healing capability and anti-freezing robustness of the iontronic film.

4. Self-healing mechanism.

In principle, the self-healing is due to the swollen of silk/Ca(II) matrix caused by water molecules and the reformation of hydrogen and coordination bonds. In detail, when water (humid air) is added onto the fracture parts, silk chains will swell up, and the viscoelasticity of the film increased, thereby leading to the physical fusing of the two separated parts. Moreover, reversible hydrogen bonds will form between the polar groups of silk side chains with both glycerol and the other polar groups of silk side chains. Owing to the dynamic bonding of the intrinsic hydrogen bonds and coordination bonds, small cracks within the film can be healed rapidly when broken bonds form again at the fractured interface.

5. Comparison of the silk-based iontronics with previous works.

Notably, Ca(II) ions and glycerol have been used separately with silk in previous works, but the performance in both cases was limited. In our article, one of the innovations is to further improve the silk-based iontronics by adding glycerol into the system, which could bring several advantages to the material. First, glycerol molecules act as the plasticizer to replace the incorporated water in protein hydration and to form intensified hydrogen bonds with the peptide matrix, resulting in the initial stabilization of α-helical structures in the films. The formation of

these strong hydrogen bonding interactions can enhance energy dissipation during the stretching process and thus increase the flexibility of iontronics film. Thus, in contrast to the previous reported silk/Ca(II) composite, the stretchability of the silk-based film (1000%, RH50) can be greatly improved with the presence of glycerol molecules as shown in the table. Second, with plentiful hydrogen bonds between glycerol and water molecules in our silk/glycerol/Ca(II) composite, the water evaporation rate could be diminished and result in a maintain of stretchability in high temperature (around 700% at 80°C). Thus, compared to the former work, our silk-based iontronics have broader temperature tolerant range. Therefore, our work combines the advantages of glycerol and Ca(II) ions, realizing the synergistic effects of these two additives for the first time.

Furthermore, we also made innovations in applications of the silk-based iontronics. As shown in the table, we report a silk-based iontronic system that can be used on both human skin and robots as a general approach for reliable robotic manipulation under harsh conditions. Thanks to the above mentioning improvements of the material, our silk-based iontronics can be used in harsh conditions, including extreme temperature and sharp objects scratching. Importantly, when coupled with a specified machine learning algorithm, our approach permits accurate human/robotic gesture identification across over 1024 classes, recoverable robotic interaction under challenging temperatures and irrespective of external damage, and grabbed-object recognition with an accuracy of 99.7%.

Supplementary Figures:

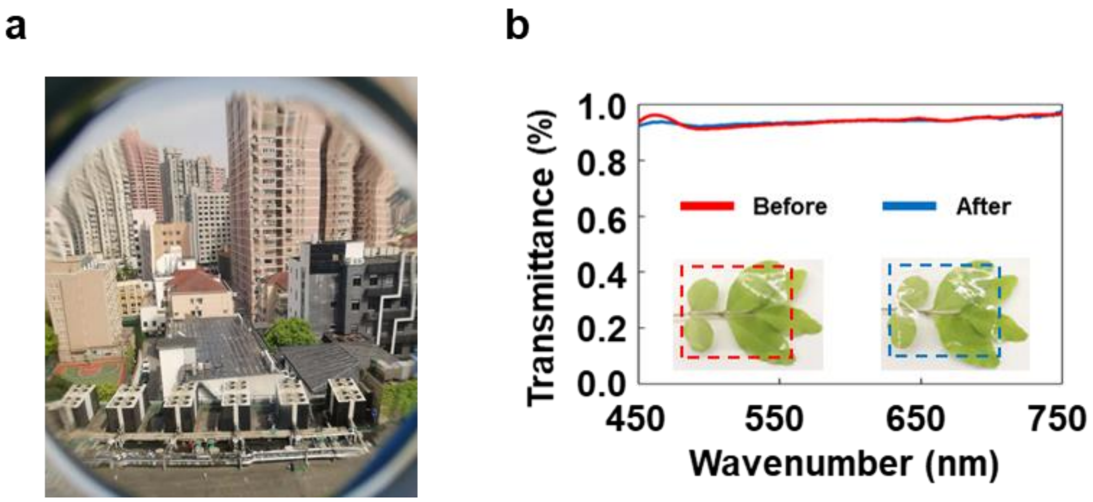

Supplementary Figure 1. Transparency of the silk-based iontronic film. a) Photographs of 1mm thick silk-based iontronics, showing high transparency. b) UV-Vis absorption spectrum of the silk-based iontronic films before and after self-healing. Inset: Images of silk-based iontronics attached on leaf before (left) and after (right) self-healing.

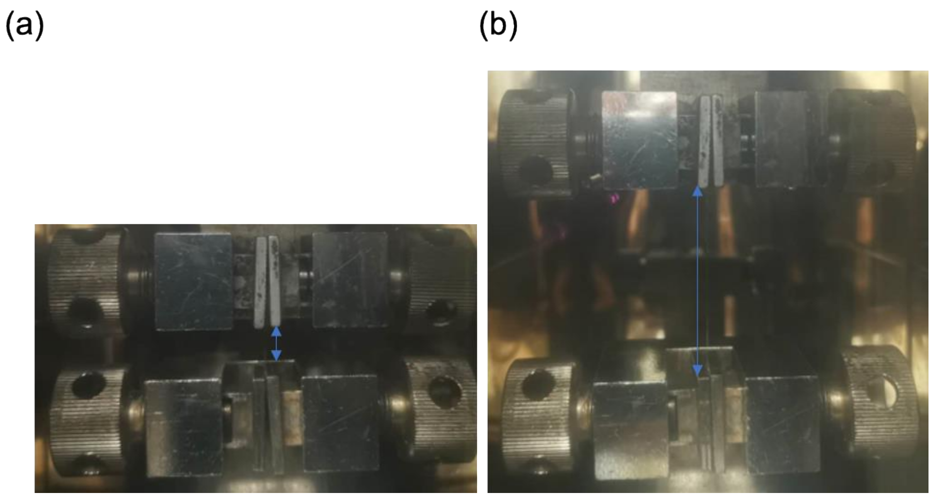

Supplementary Figure 2. Photographs of stretchable performance of silk-based iontronics at harsh temperatures (-40–120°C).

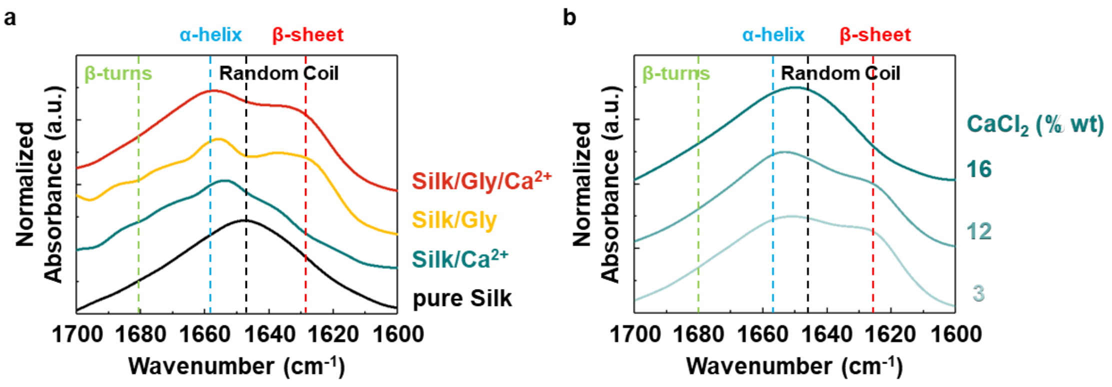

Supplementary Figure 3. Fourier-transformed infrared spectroscopy (FTIR) of the silk-based iontronic films. a) FTIR spectra of silk-based films with different compositions, including silk/glycerol/Ca(II), silk/glycerol, silk/ Ca(II) and pure silk. b) FTIR spectra of silk-based iontronic films with different Ca(II) concentration (3 wt%, 12 wt% and 16 wt%).

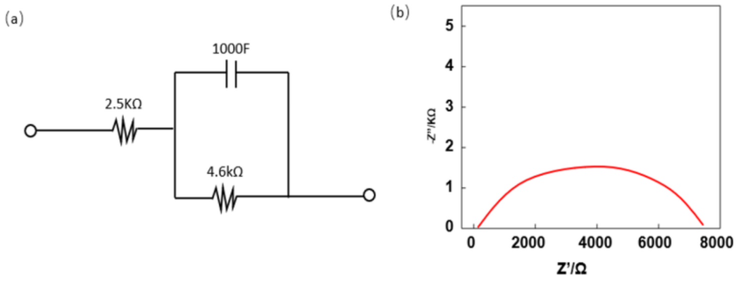

Supplementary Figure 4. (a) Testing circuit and (b) Nyquist plot of silk-based iontronics.

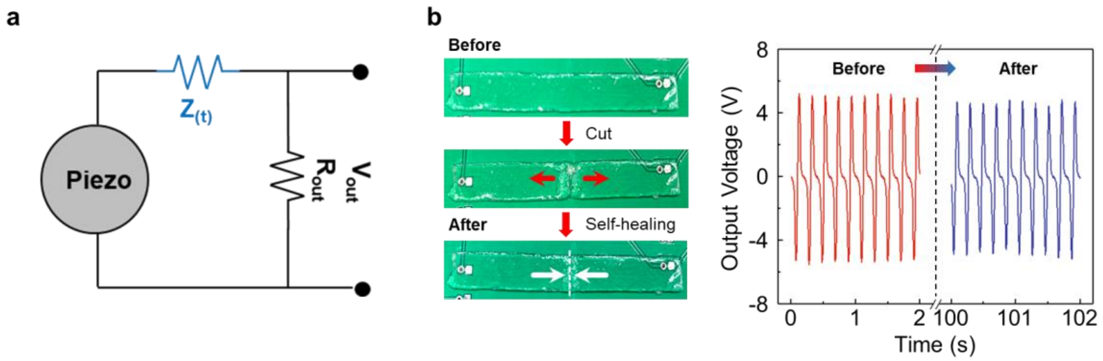

Supplementary Figure 5. Self-healable conductive wires. (a) Schematic illustration of silk-based iontronic film acting as conductive wire in a piezoelectric circuit. (b) Output voltage before and after self-healing in the piezoelectric circuit.

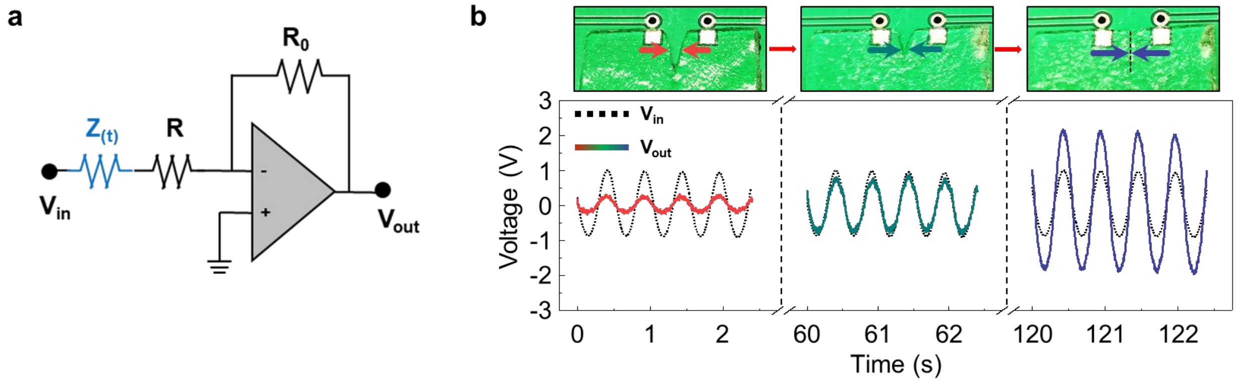

Supplementary Figure 6. Reconfigurable component for amplification adjustment. (a) Schematic illustration of a circuit design for an analog amplifier. The blue color resistance denotes the self-healable silk-based iontronics. (b) Changes in output gain of the amplifier by the self-healing of silk-based iontronics. Inset: photographs of silk-based iontronics during the self-healing process.

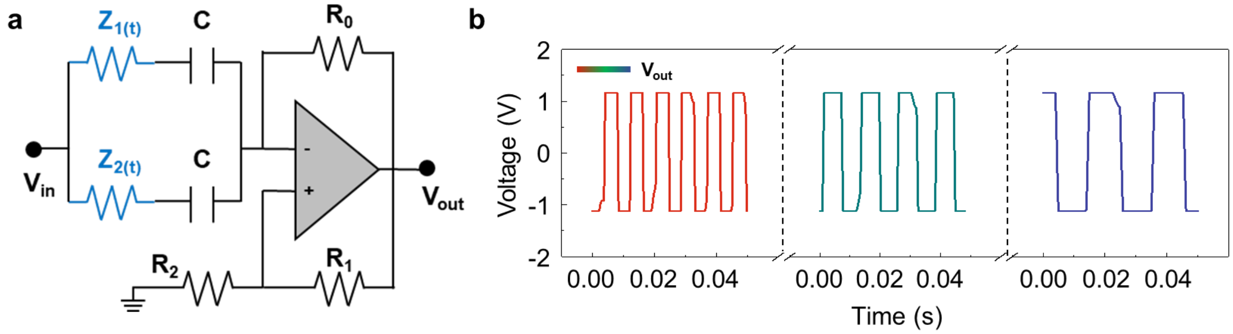

Supplementary Figure 7. Reconfigurable component for frequency adjustment. (a) Schematic illustration of the circuit design for the waveform generator. The blue color resistances denote the self-healable silk-based iontronics. (b) Changes in the resonant frequency of the waveform generator induced by the self-healing of silk-based iontronics.

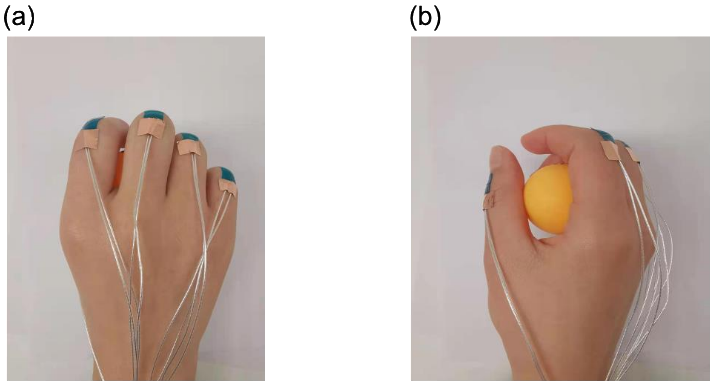

Supplementary Figure 8. Images of human hand, mounted with silk-based iontronics, grabbing ping-pong ball.

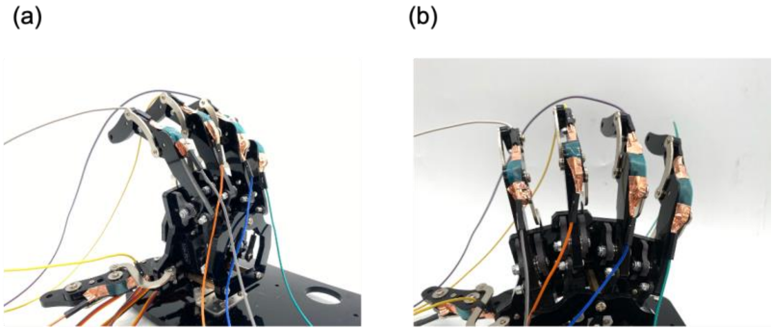

Supplementary Figure 9. Images of robotic hand, mounted with silk-based iontronics.

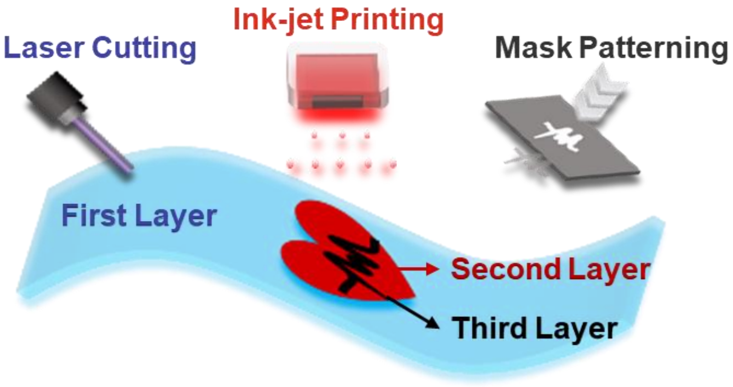

Supplementary Figure 10. Potential fabrication and modification process of silk-based iontronics.

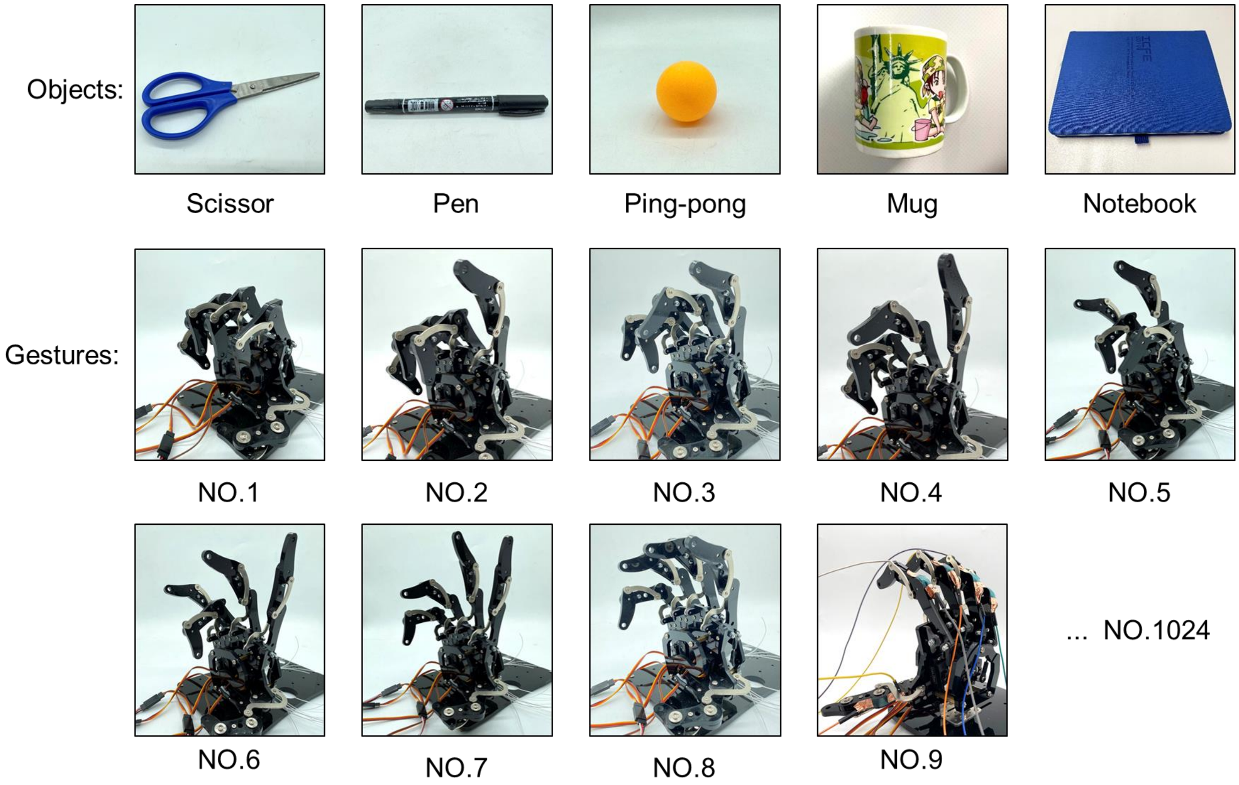

Supplementary Figure 11. Target objects and robotic hand gestures for building the dataset.

In total, 5 objects and 1024 gestures are used in our dataset, whose images are shown here.

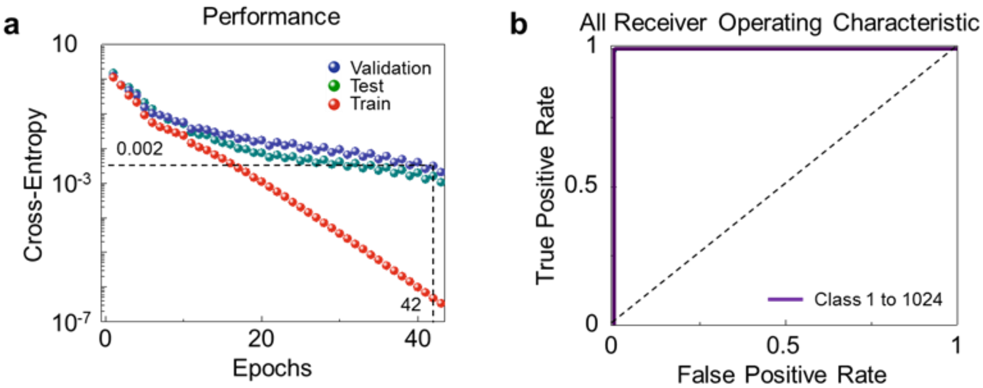

Supplementary Figure 12. The performance characterization of the pattern recognition ANN for learning robotic hand gestures at high temperature, demonstrating minimum cross-entropy at 42 epochs and true positive for all classes (1024 types).
